# Traumatic abdominal wall hernia in two adults: a case series

**DOI:** 10.4076/1752-1947-3-7324

**Published:** 2009-06-30

**Authors:** Nitin Agarwal, Sunil Kumar, Mohit Kumar Joshi, Mriganka Sekhar Sharma

**Affiliations:** 1 Department of Surgery, University College of Medical Sciences and Guru Teg Bahadur Hospital, Vivek Vihar, Delhi 110095, India

## Abstract

**Introduction:**

Traumatic hernia of the abdominal wall is a rare entity. A large proportion of reported cases are in children with a particular type of injury, i.e. from a handlebar injury. In adults, the presentation can vary substantially and the diagnosis is difficult. We present two cases in adults, with widely varying presentations and management.

**Case presentations:**

A 40-year-old woman from rural north India presented with a low-velocity blunt injury to the lower abdomen. She was attacked by a bull. She had a clinically evident abdominal fascial disruption with intact skin, and was hemodynamically stable. An emergency mesh repair of the defect was performed, and she recovered well.

A 38-year-old man from rural north India presented with blunt trauma to the abdomen following a motor vehicle accident. He was stable, with a central abdominal parietal wall swelling and bruising. A computed tomography scan revealed herniation of bowel loops in the area with minor intra-abdominal injuries. A laparotomy, resection-anastomosis of the ischemic bowel, and primary repair of the defect was performed and he recovered well.

**Conclusion:**

Following blunt abdominal trauma, particularly high-velocity injuries, a high index of suspicion must be reserved for parietal wall swellings, as missed hernias in this setting have a high risk of strangulation. Computed tomography is the best aid to diagnosis. Management of each case needs to be individualized.

## Introduction

Traumatic disruption of the abdominal wall is very rare, with only about 50 reports worldwide, and only one from India [1]. Not surprisingly, many cases have been reported in children, given their weaker parietal wall and more elastic skin [2]-[4]. The diagnosis is rarely straightforward, and management can vary substantially due to differences in presentation [5]. We report two cases of adults with different presentations of traumatic abdominal wall hernia, with a brief review of the literature.

## Case presentation 1

A 40-year-old woman of north Indian origin was brought to the surgical emergency room 2 hours after being attacked by a bull near her home. The animal had struck her with its horns over her right lower abdomen. The patient complained of pain and swelling over the involved area. On examination, her vital signs were normal. There was no associated head, chest, pelvic, or limb injury. A bruise and a visible, ill-defined swelling over the right lumbar and umbilical regions were seen. On palpation, the area was tender, and the swelling decreased on gentle pressure, with gurgling sounds. A defect of 8 cm in maximum diameter could be palpated with irregular, ill-defined margins. On asking the patient to cough, bowel loops could be palpated through the defect. Since the patient was hemodynamically stable and the diagnosis clinically apparent, a transverse laparotomy was performed incorporating the site of injury. An irregular defect was found involving all subcutaneous layers of the abdominal wall, and was repaired with a preperitoneal mesh. There was no associated intra-abdominal injury. The patient's recovery was uneventful and she was discharged on the 3rd postoperative day. She had no recurrence after 1 year of follow-up.

## Case presentation 2

**Figure 1:**
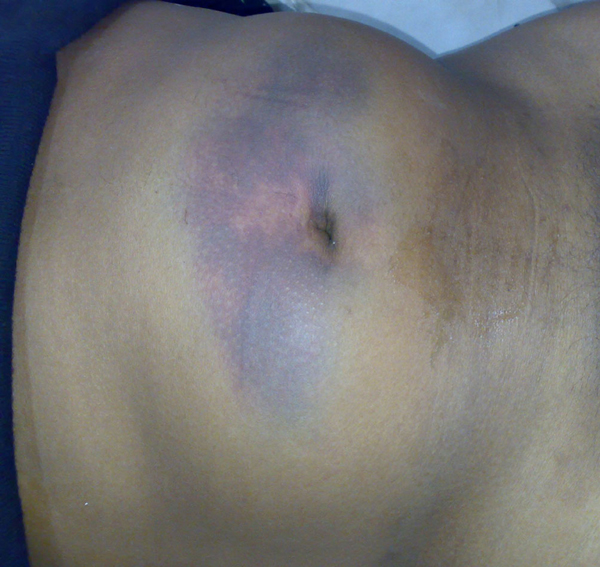
**Parietal swelling with bruising after motor vehicle accident**.

**Figure 2:**
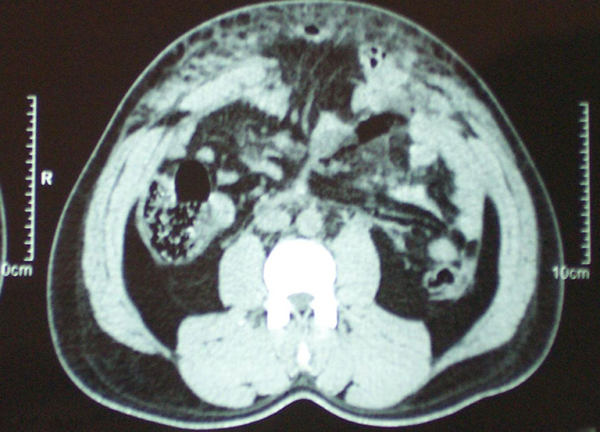
**Contrast enhanced computed tomography showing traumatic hernia in the same patient**. Supra-umbilical defect in the midline anterior abdominal wall (maximum diameter 5 cm), with herniation of small bowel loops and mesentery through it, with extensive interstitial edema.

A 38-year-old north Indian man was brought to the emergency department of our hospital after suffering a head-on collision while driving his car. On examination, he was conscious, oriented, and, in severe pain. His vital signs were normal, except for tachycardia. There were no signs of head, chest, pelvic, or limb injury. A visible swelling was present in the umbilical and epigastric regions with overlying bruising of the skin (Figure 1). On palpation, it was tender, firm, non-pulsatile, and non-reducible, but there was a slight increase in size over the next hour. The patient was triaged as a blunt trauma abdomen - a probable steering wheel injury, hemodynamically stable, with probable rectus hematoma. Expectant treatment was started and a computed tomography (CT) scan was performed. The CT scan (Figure 2) revealed a supra-umbilical defect in the midline anterior abdominal wall (maximum diameter 5 cm), with herniation of bowel loops and mesentery through it, with extensive interstitial edema. There was also associated free fluid in the peritoneal cavity, and a minor splenic laceration. Anticipating strangulation of the bowel, the abdomen was explored through a midline incision. There were 2-3 loops of edematous jejunum and mesentery in the subcutaneous space, herniating from a full-thickness defect in the abdominal wall. There was also a mesenteric hematoma with loss of vitality of the corresponding jejunal segment (one foot). A resection-anastomosis was performed. No other solid organ injuries were seen. The defect was repaired with an interrupted monofilament polypropylene suture without tension. The patient's wound healed without complications and was discharged on the 6th day. He was asymptomatic, 1 month after the operation.

## Discussion

Traumatic hernia of the abdominal wall is a rare injury. It is caused by trauma sufficient to disrupt fascial layers, but not the elastic skin. Many mechanisms have been thought to cause traumatic abdominal wall hernias. The most common appears to be bicycle handlebar injury, especially in children [2]-[4,6]. Wood et al. [6] attempted to classify these mechanisms into three types, namely: 1) small lower quadrant defects such as a handlebar injuries; 2) larger abdominal wall defects such as motor accidents; and 3) intra-abdominal herniations such as a deceleration injury. Recently, Netto *et al.*[7] carried out a retrospective review of 34 patients with traumatic abdominal wall hernia, and made three recommendations. First, they concluded that the mechanism of injury should be considered when deciding on operative intervention. Second, clinically apparent hernias often have associated injuries and warrant urgent laparotomy. Finally, occult hernias may be managed expectantly.

In both of our patients, the hernia was clinically apparent with a palpable lump, but no associated injury was seen in the first case. CT scan is unequivocally the best modality for diagnosis [8]-[10]. It is also useful for identification of associated injuries. With Netto et al's recommendations (*vide supra*), we could have performed a CT scan and avoided an emergency operation in the first patient. The hernia repair could have been carried out at a later date, but lack of timely availability of a CT scanner and the rarity of the condition confounded our decision. Recently, Matsuo and colleagues have also reported successful conservative management of abdominal hernia caused by handlebar injury, using a cloth corset [11]. Their decision was again aided by a CT scan which did not reveal any intra-abdominal injury. Hence, decisions with such patients are best made on an individual basis.

Both mesh repair as well as primary repair have been successfully performed for treatment of traumatic hernia [2]-[5]. As there appears to be no consensus on this issue, one may conclude that low-velocity injuries lead to less tissue necrosis, and a mesh can be used. When high-velocity injuries are present as in motor vehicle accidents, mesh may be avoided because of the high risk of infection, unless there is greater tissue loss [5]. Treatment based on the merits of each case would again be the most prudent approach. A high index of clinical suspicion is also essential, as an accompanying hematoma often confounds the diagnosis [10].

## Conclusion

Following blunt abdominal trauma, particularly high-velocity injuries, a high index of suspicion must be reserved for parietal wall swellings, as missed hernias in this setting have a high risk of strangulation. Computed tomography is the best aid to diagnosis. Management of each case needs to be individualized.

## Consent

Written informed consent was obtained from the patients for publication of this case report and any accompanying images. A copy of the written consent is available for review by the Editor-in-Chief of this journal.

## Competing interests

The authors declare that they have no competing interests.

## Authors' contribution

NA analyzed and interpreted the patient data regarding traumatic hernia and its presentation. SK and MKJ were major contributors in writing the manuscript. MSS also contributed to writing the manuscript, and performed the first operation. All authors read and approved the final manuscript.
